# Flexible Mesh-Structured Single-Walled Carbon Nanotube Thermoelectric Generators with Enhanced Heat Dissipation for Wearable Applications

**DOI:** 10.3390/mi17010139

**Published:** 2026-01-22

**Authors:** Hiroto Nakayama, Takuya Amezawa, Yuta Asano, Shuya Ochiai, Keisuke Uchida, Yuto Nakazawa, Masayuki Takashiri

**Affiliations:** 1 Department of Materials Science, Tokai University, Hiratsuka 259-1292, Kanagawa, Japan; 2 Department of Applied Chemistry, Tokai University, Hiratsuka 259-1292, Kanagawa, Japan

**Keywords:** thermoelectric generator, heat dissipation, dip-coated single-walled carbon nanotubes (SWCNTs), mesh sheets, wearable sensors

## Abstract

Thermoelectric generators (TEGs) based on single-walled carbon nanotubes (SWCNTs) offer a promising approach for powering sensors in wearable systems. However, achieving high performance remains challenging because the high thermal conductivity of SWCNTs limits the temperature gradient within the device. We previously developed flexible SWCNT-TEGs with enhanced heat dissipation by dip-coating SWCNTs onto mesh sheets; however, their performance in real wearable environments had not been evaluated. In this study, we demonstrate the practical operation of these SWCNT-TEGs under conditions such as fingertip contact and cap-based wear. The output voltage increased proportionally with the number of fingers touching the device, and a stable voltage of 6.1 mV was obtained when the TEG was mounted on a cap and worn outdoors at 7 °C. These findings highlight the promising potential of flexible SWCNT-TEGs as power sources for next-generation wearable technologies, including human–computer interaction and health monitoring.

## 1. Introduction

Advances in science and technology in recent years have greatly improved the quality of life. Driven by the development of ubiquitous computing, the Internet of Things (IoT) is expected to play a central role in future technological innovation [1,2,3,4]. IoT devices rely on distributed sensors capable of continuously acquiring and transmitting information. However, a major challenge in their deployment is securing a stable and maintenance-free power supply. Energy-harvesting technologies, which convert small amounts of ambient energy—such as vibration, light, and radio-frequency waves—into electricity, have therefore attracted considerable attention [5,6,7,8,9,10,11,12,13].

Thermoelectric power generation is particularly promising because it enables electricity production from ubiquitous heat sources, including industrial waste heat and natural thermal gradients [14,15,16,17,18,19,20]. Wearable thermoelectric generators (TEGs) can further utilize body heat as an energy source, enabling self-powered health-monitoring devices, motion-control sensors, and human–computer interaction tools [21,22,23,24,25].

Single-walled carbon nanotubes (SWCNTs) have emerged as next-generation thermoelectric materials for wearable TEGs owing to their flexibility, mechanical robustness, and solution processability [26,27,28,29]. SWCNTs exhibit either metallic or semiconducting behavior depending on their chirality, and semiconducting SWCNT networks can be assembled into thin films suitable for flexible TEGs [30]. Although pristine SWCNTs typically show *p*-type behavior, *n*-type SWCNTs can be obtained through chemical doping [31,32,33]. However, the intrinsically high thermal conductivity of the SWCNT films makes it difficult to establish a sufficient temperature gradient across the films, which limits their thermoelectric performance. This challenge has motivated the development of novel SWCNT-based films with reduced thermal conductivity or enhanced thermal isolation [34,35].

Two strategies have been explored to address this issue. The first involves fabricating composite films by incorporating polymers or inorganic additives to suppress thermal transport [36,37,38,39,40]. For example, Nakazawa et al. demonstrated that blending SWCNTs with an inorganic-modified acrylic emulsion reduced the thermal conductivity to one-fourth that of pristine films [41]. The second strategy focuses on structural control, such as introducing porosity or defects to scatter phonons [42,43,44,45,46,47]. Yang et al. reported that porous CNT sponges exhibit significantly reduced thermal conductivity due to enhanced phonon scattering [48].

Building on these developments, our previous work introduced a new approach by engineering the film–substrate architecture rather than modifying the SWCNT network itself. We fabricated *p*-type SWCNT films by dip-coating mesh substrates with SWCNT ink, resulting in highly flexible films with enhanced heat dissipation [49]. The TEGs based on these mesh-structured films exhibited larger temperature differences than those based on conventional SWCNT films when evaluated on a heater. Furthermore, stable *n*-type films with the same architecture were successfully obtained through cationic-surfactant doping followed by fluoropolymer coating [50].

However, all previous evaluations—including our own—were conducted under controlled laboratory conditions using heaters, and no studies have examined the performance of mesh-structured SWCNT TEGs in actual wearable environments, where heat transfer is strongly influenced by skin contact, airflow, and ambient temperature. This gap is critical because wearable applications require TEGs to operate under dynamic and unpredictable thermal conditions, which differ substantially from heater-based testing.

In this study, we address this gap by evaluating the performance of mesh-structured SWCNT TEGs in two representative wearable scenarios: fingertip contact and wearing a cap. In the fingertip test, the output voltage increased proportionally with the number of fingers touching the device, demonstrating a clear relationship between contact area and heat input. In the cap-wearing test, the TEG mounted on the forehead generated a stable output voltage of 6.1 mV under outdoor conditions. These findings provide the first demonstration of the practical thermoelectric behavior of mesh-structured SWCNT films in real wearable environments and highlight their potential for self-powered IoT applications, including human–computer interaction and health monitoring.

## 2. Materials and Methods

**Figure 1:**
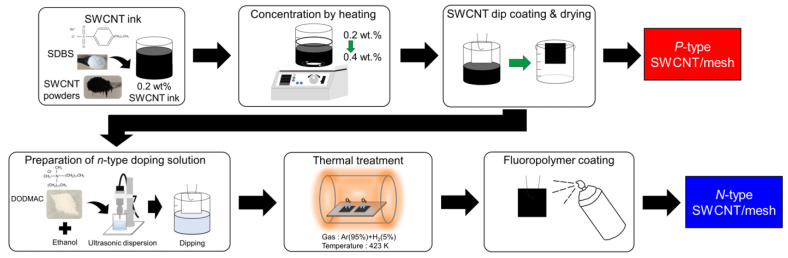
Fabrication of dip-coated single-walled carbon nanotube (SWCNT) film onto PPS mesh sheet.

The detailed fabrication processes of the SWCNT films are described in our previous reports [49,50] and a brief process is shown in Figure 1. The SWCNTs used in this study were synthesized by the super-growth method (SG-CNTs) [51]. The SWCNT ink was provided by Zeon Corporation (Tokyo, Japan). This ink was prepared by dispersing 0.2 wt.% SWCNTs and 1.0 wt.% of the anionic surfactant (sodium dodecylbenzenesulfonate (SDBS, C, FUJIFILM Wako Pure Chemical, Osaka, Japan) in deionized water. To increase the viscosity of the SWCNT inks, the concentrations of the SWCNTs and SDBS were increased to 0.4 and 2.0 wt.%, respectively, by heating the ink in an open beaker at approximately 350 K for 12 h to evaporate water and thereby concentrate the dispersion. The mesh sheets were made of polyphenylene sulfide (PPS) and had a 100 μm aperture and a 34 μm wire diameter (Clever Co., Toyohashi, Japan). To prepare *p*-type SWCNT films, the PPS mesh sheet was immersed for 2 s and withdrawn at a speed of 8 mm/s. After removing the sheet from the SWCNT ink, it was dried in ambient air at approximately 300 K for 24 h. This dipping process with SWCNT ink was repeated one more time to ensure sufficient adhesion of the SWCNTs to the mesh sheet surface. To prepare the *n*-type SWCNT films, the completed *p*-type SWCNT films without an SDBS-removal step were dipped into a cationic surfactant solution, which was 2.5 wt.% of dimethyldioctadecylammonium chloride (DODMAC, FUJIFILM Wako Pure Chemical, Osaka, Japan) in ethanol, for 2 s and withdrawn at a speed of 8 mm/s. After the SWCNT film was removed from the DODMAC solution, it was dried in ambient air at approximately 300 K for 1 h. The dipping process in the DODMAC solution was repeated one more time to ensure sufficient DODMAC adhesion to the SWCNT surface. To exhibit *n*-type properties, the SWCNT films were subjected to thermal treatment at 423 K for 1 h in an electric furnace under a flowing 95% Ar–5% H_2_ atmosphere (1 slm), with the furnace temperature increased at a rate of 4 K/min. Finally, to maintain the *n*-type properties for a long time, a fluoropolymer (Superhydrophobic, FK, Shijonawate, Japan) coating was applied to the *n*-type SWCNT films.

The microstructure of the dip-coated SWCNT/mesh films was observed using field-emission scanning electron microscopy (FE-SEM; S-4800, Hitachi, Ltd., Tokyo, Japan). The Seebeck coefficient (*S*) of the films was measured at approximately 300 K with an accuracy of ±5% using home-made equipment, based on our previous study [49,50]. The electrical conductivity (*σ*) was measured at a temperature of approximately 300 K using the four-point probe method (RT-70V, Napson, Tokyo, Japan) with an accuracy of ±3%. The Seebeck coefficient and electrical conductivity were measured thrice at different positions on each sample, and the obtained values were averaged. The power factor *σS*^2^ was calculated based on the measured Seebeck coefficient and electrical conductivity values.

## 3. Results and Discussion

### 3.1. Structural, Thermoelectric Properties, and Heat Dissipation of Single-Walled Carbon Nanotube (SWCNT) Films

**Figure 2:**
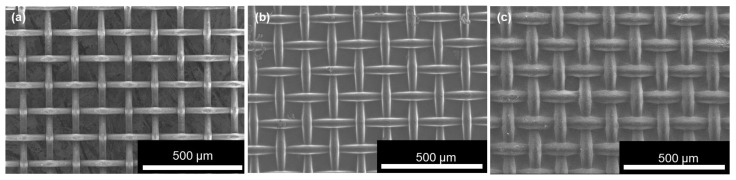
Microstructure of dip-coated SWCNT/mesh films. Reprinted from Ref. [50]. (**a**) Only PPS mesh sheet, (**b**) *p*-type dip-coated SWCNT/mesh film, and (**c**) *n*-type dip-coated SWCNT/mesh film.

Figure 2 presents the microstructure of the dip-coated SWCNT/mesh films [50]. For reference, an uncoated PPS mesh sheet is shown in Figure 2a. The mesh wire diameter was approximately 35 µm and the opening size was 100 µm square, consistent with the product specifications. Figure 2b shows the *p*-type dip-coated SWCNT/mesh films, in which the wire diameter increased to approximately 40 µm and the opening size decreased, suggesting that SWCNTs were uniformly deposited onto the PPS mesh while maintaining the mesh openings. An additional higher-magnification FE-SEM image is provided in the Supplementary Materials to more clearly visualize the SWCNTs (Figure S1). Figure 2c shows the *n*-type dip-coated SWCNT/mesh films. After coating DODMAC and fluoropolymer onto the SWCNT surfaces, both the wire diameter and opening size increased further. This confirms that the DODMAC and fluoropolymer layers were successfully deposited on the SWCNT layer, as supported by our previous study in which chlorine from DODMAC and fluorine from the fluoropolymer were detected on the SWCNT surface [50].

Table 1 summarizes the thermoelectric properties of the *p*- and *n*-type dip-coated SWCNT/mesh films, along with those of SWCNT films without openings fabricated from the same SG-CNTs via vacuum filtration and drop casting [30,41]. The absolute Seebeck coefficients of the *p*-type dip-coated films were higher than those of the *n*-type films, whereas their electrical conductivities were lower. Consequently, both film types exhibited comparable power factors. Thermal conductivity could not be measured because the laser beam used in the noncontact laser-spot periodic-heating radiation calorimetry method passed through the mesh openings. However, the effective thermal conductivity of the SWCNT mesh/films was evaluated using the comparative cut-bar method. Detailed experimental and measurement procedures are provided in the Supplementary Materials (Figure S2). The *p*-type and *n*-type SWCNT mesh/films exhibited effective thermal conductivities of 3.6 W/(m·K) and 6.0 W/(m·K), respectively, both of which are lower than those of the corresponding SWCNT films without openings. The higher thermal conductivity observed for the *n*-type mesh/film compared with the *p*-type mesh/film is attributed to the reduced mesh openings caused by the DODMAC and fluoropolymer coatings, as evidenced by the FE-SEM images in Figure 2. For the *p*-type films, the Seebeck coefficients were nearly identical, but the film without openings showed higher electrical conductivity and thus a higher power factor. In contrast, in the *n*-type films, the electrical conductivities were nearly the same, whereas the film without openings exhibited a higher Seebeck coefficient and a correspondingly higher power factor.

**Figure 3:**
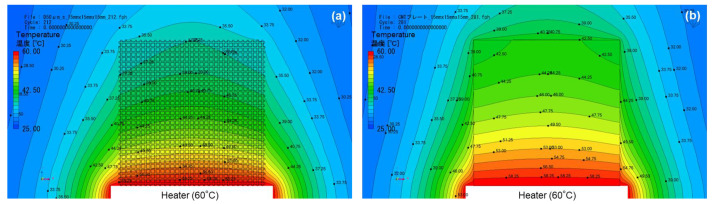
Temperature distributions from CFD simulations for (**a**) *p*-type dip-coated SWCNT/mesh film and (**b**) *p*-type SWCNT film without openings. In both cases, the bottom surface of the film was heated to 60 °C, and natural convection was considered in the simulation.

To elucidate the influence of mesh geometry on heat transport under natural convection, we conducted computational fluid dynamics (CFD). The details of the computational modeling are provided in the Supplementary Materials (Figure S3). Figure 3 presents the temperature distributions of the *p*-type dip-coated SWCNT/mesh film when the bottom surface of the film was heated to 60 °C, while the *p*-type SWCNT film without openings served as a reference for comparison. Although both films shared the same macroscopic dimensions (45 mm × 45 mm) and were subjected to identical heating conditions at the lower edge (60 °C), their temperature distributions diverged significantly. The SWCNT/mesh film exhibited a larger temperature drop of 21.7 K from bottom to top, whereas the SWCNT film without openings showed a smaller drop of 19.2 K. This difference cannot be attributed solely to variations in material properties, as both films consisted of *p*-type SWCNT networks. The openings facilitated enhanced convective heat loss from the film surface. Natural convection cells could more easily develop around and through the mesh, increasing the effective heat transfer coefficient and further lowering the temperature near the upper edge. In contrast, the continuous SWCNT film suppressed such convective penetration and maintained more uniform lateral heat spreading, resulting in a smaller temperature gradient. Under forced convection, the temperature difference between the lower and upper edges of both films was expected to become even larger, because the increased airflow would further enhance heat removal from the film surfaces—particularly through the openings in the mesh structure. Moreover, similar tendencies were anticipated even when the thermoelectric properties of the films were converted to *n*-type, as the dominant factor governing the temperature distribution was the geometric configuration rather than the carrier type of the SWCNT network. Therefore, the larger temperature difference achieved with the SWCNT/mesh films suggests that TEGs fabricated with this structure can be expected to exhibit higher output voltages.

### 3.2. Fabrication and Performance of SWCNT-TEGs by Touching Fingertips

**Figure 4:**
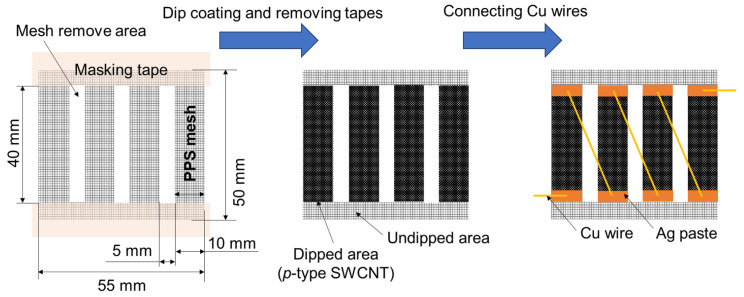
Fabrication process of TEG using *p*-type dip-coated SWCNT/mesh films for touching fingertips.

Figure 4 summarizes the fabrication process of a fingertip-responsive TEG based on dip-coated SWCNT/mesh films. To ensure a linear voltage response proportional to the number of touching fingers, only *p*-type dip-coated films were used, because the Seebeck coefficients of *p*- and *n*-type films differed substantially (Table 1). A PPS mesh sheet (100 µm aperture, 34 µm wire diameter) was first cut into a 55 mm × 50 mm piece. In addition, three rectangular areas located near the center of the sheet—each measuring 40 mm × 5 mm—were removed to obtain the final device geometry. Masking tape was then applied to define the coating regions. Four SWCNT-coated areas (40 mm × 10 mm each) were patterned in horizontal rows with 5 mm spacing. The SWCNT ink formulation and dip-coating procedure followed those described in Section 2. After drying, the masking tape was removed, and thin copper wires were used to connect the adjacent coated regions in series.

**Figure 5:**
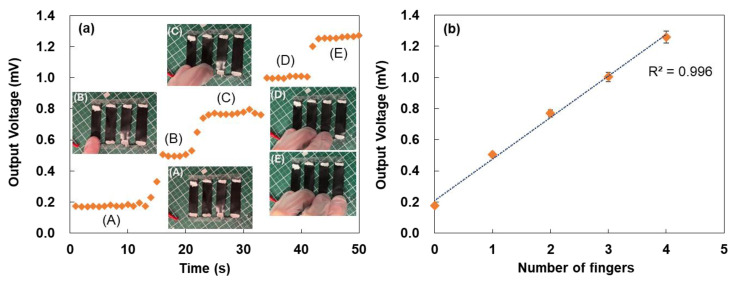
(**a**) Output voltage of the TEG under different fingertip-touch conditions, measured while the TEG was placed on a flat desk surface. Insets show images of the device touched by (A) no finger, (B) one finger, (C) two fingers, (D) three fingers, and (E) four fingers. The touch duration in panel (B) was shorter than in the other conditions due to an experimental handling error; this did not affect the intended qualitative comparison. (**b**) Relationship between the number of fingers and output voltage measured from the TEG.

Figure 5 shows the voltage response of the TEG under various fingertip contact conditions, measured while the TEG was placed on a flat desk surface. As shown in Figure 5a, a small baseline voltage of 0.17 mV was observed even without fingertip contact, likely due to a slight temperature gradient generated by natural convection around the device surface. Similar convection-induced voltages have been reported in flexible thermoelectric systems, indicating that minimal environmental fluctuations can produce measurable signals [52]. When a fingertip touched the SWCNT-coated area, the output voltage rapidly increased to 0.50 mV with an initial rate of 0.11 mV/s. This sharp increase reflects efficient thermal coupling between the human skin and the SWCNT film, which exhibits high in-plane thermal conductivity and rapid heat spreading. After the transient response, the voltage reached a steady state, indicating rapid thermal equilibration between the fingertip and the TEG surface. As the number of fingers increased, the output voltage exhibited an almost linear increase. This behavior suggests that the effective heat input scales proportionally with the contact area, consistent with trends reported for CNT-based tactile thermal sensors [53]. The relationship between the number of fingers and output voltage measured from the TEG is shown in Figure 5b. The rate of increase and steady-state stability remained nearly constant regardless of the finger count, demonstrating robust thermal sensitivity, and this linearity was further supported by an excellent correlation coefficient (R^2^ = 0.996).

These characteristics are highly advantageous for touch-sensing applications [54,55,56], as they indicate predictable and reproducible thermoelectric signal generation. The distinct voltage separation observed for different numbers of contacting fingertips enabled unambiguous signal discrimination. Such discrete voltage levels could be readily mapped to predefined input commands; as an example, the voltage values associated with the inset images Figure 5a(B)–(E) could be assigned to the digits “0,” “1,” “7,” and “9,” respectively. This simple encoding scheme suggests the feasibility of using the TEG as a self-powered input interface for emergency-call sequences such as “110,” “119,” or “171.” This concept is consistent with recent developments in self-powered human–machine interfaces, in which thermoelectric outputs are exploited to encode user intent without relying on external power supplies [57].

**Figure 6:**
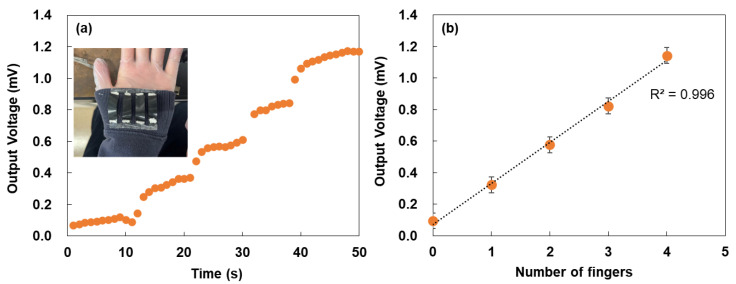
(**a**) Output voltage of the TEG under different fingertip-touch conditions, measured while the TEG was attached to the clothing. Inset shows image of TEG attached to clothing cuffs in each fingertip condition. (**b**) Relationship between the number of fingers and output voltage measured from the TEG.

To evaluate wearable applicability, the TEG was attached to the cuff of a garment, and the output voltage was measured, as shown in Figure 6a. The voltage increased proportionally with the number of fingers touching the device, similar to the desk-mounted condition. The voltage levels were nearly identical in both configurations, indicating that thermal transport through the fabric did not significantly attenuate heat input. This observation is consistent with previous reports on textile-integrated thermoelectric devices [58]. However, slight delays and increased noise were observed in the wearable configuration. These effects may have arisen from thermal buffering by the fabric, variations in contact pressure, or micro-movements during finger placement. To analyze the behavior in more detail, the relationship between the number of fingers and the output voltage measured from the TEG is shown in Figure 6b. Although the error bars were larger than those in Figure 5b, both the voltage rise and the steady-state level showed minimal dependence on the number of fingers, indicating robust thermal sensitivity. This linear behavior was further confirmed by the high correlation coefficient (R^2^ = 0.996). Future improvements could include reducing the thermal resistance between the TEG and the fabric, enhancing mechanical fixation, or incorporating materials with higher thermal diffusivity. Addressing these factors would improve the responsiveness and reliability of the TEG for practical applications in wearable sensing.

### 3.3. Fabrication and Performance of SWCNT-TEGs by Wearing Cap

**Figure 7:**
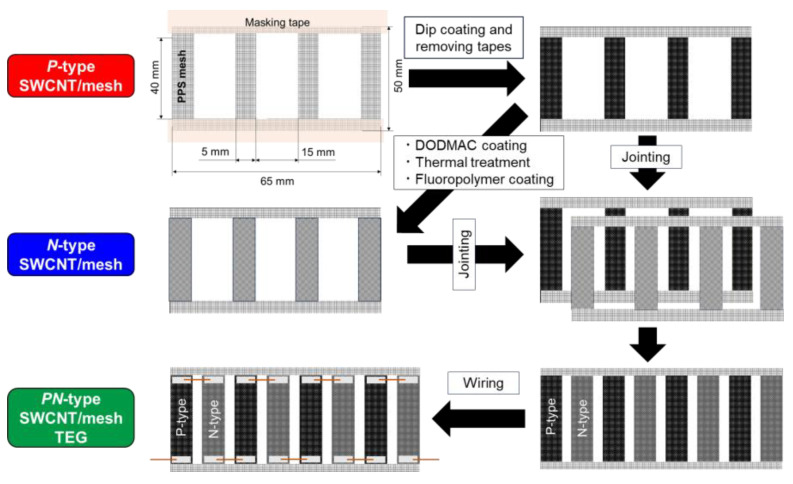
Fabrication process of TEG using dip-coated SWCNT/mesh films for wearing caps.

Figure 7 illustrates the fabrication process of the SWCNT-TEG designed for wearable operation on a cap. Unlike the fingertip-responsive TEG, which required a linear voltage response, the wearable device prioritized a higher output voltage. Therefore, both *p*- and *n*-type dip-coated films were used to form a TEG. Two PPS mesh sheets (100 µm aperture, 34 µm wire diameter), each cut into a 65 mm × 50 mm piece, were prepared for subsequent coating. In addition, three rectangular sections located near the center of each sheet—each measuring 40 mm × 15 mm—were removed to obtain the final device geometry. This cutting process resulted in 40 mm × 5 mm PPS-mesh strips between the openings, and these remaining strips served as the coating regions. Masking tape was applied to define a 40 mm × 5 mm area within each strip, and four SWCNT-coated regions were patterned in horizontal rows with 15 mm spacing. Both PPS mesh sheets were then dip-coated with SWCNT ink to form *p*-type SWCNT/mesh films. After drying, the masking tape was removed. Subsequently, one of the two *p*-type films was converted to *n*-type using the procedure described in Section 2. After preparing the *p*- and *n*-type films, they were arranged alternately at uniform intervals to construct the *p*–*n* junction structure. Thin copper wires were used to connect adjacent *p*- and *n*-type regions at the top and bottom edges, forming a series-connected TEG.

**Figure 8:**
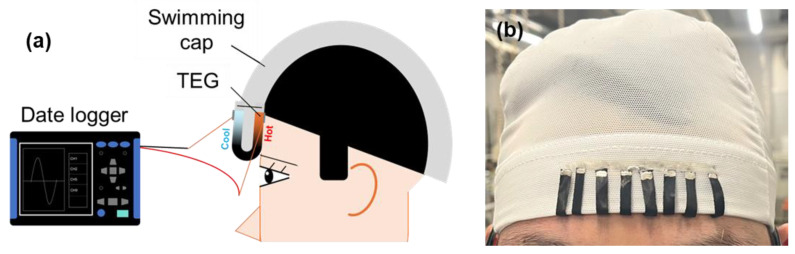
(**a**) Schematic diagram of fabricated TEG sewn onto cap and attached to human body and (**b**) image of person wearing a cap with TEG.

Figure 8a shows a TEG sewn onto a commercially available swimming cap. Approximately half of the device contacted the wearer’s forehead (36.5 °C), while the remaining half was exposed to ambient air, enabling power generation from the resulting temperature gradient. To prevent any interference between the skin and the thermoelectric elements or Cu wire connections, the hot-side surface of the TEG was covered with a polyimide tape, which served as a protective insulating layer. The output voltage was measured using a data logger (LR8432, HIOKI, Nagano, Japan), and the temperature distribution was monitored using a thermographic camera (OPTRIS, Berlin, Germany). An image of the wearable configuration is shown in Figure 8b.

**Figure 9:**
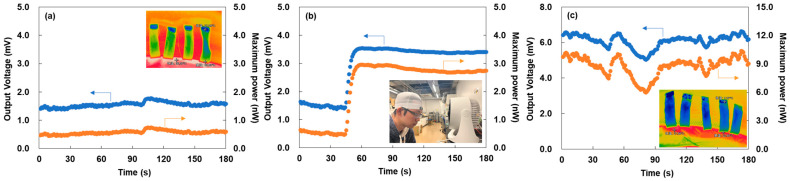
Performance of the wearable TEG under different environmental conditions: (**a**) temperature of 28 °C indoors with no wind, (**b**) temperature of 28 °C indoors with a wind speed of 3 m/s, and (**c**) temperature of 7 °C outdoors with almost no wind.

The performance of the wearable TEG under different environmental conditions is shown in Figure 9. The maximum power, *P_max_*, was calculated from the output voltage *V* and the measured total electrical resistance of the TEG, *R_total_*, as follows: *P_max_* = *V*^2^/4*R_total_*, where the *R_total_* of the wearable TEG used in this study was 1060 Ω. Because the wearable TEG incorporates multiple *p–n* junctions and copper-wire connections, the contact resistance has the potential to vary depending on the fabrication conditions. Although such variations do not affect the measured output voltage, they directly influence the total electrical resistance. Figure 9a shows the output voltage and maximum power measured indoors at 28 °C under windless conditions. The wearable TEG exhibited a stable output voltage of 1.4 mV and a maximum power of 0.5 nW. Using the Seebeck coefficients listed in Table 1, the corresponding temperature difference across the TEG was estimated to be 3.9 K. This value is in close agreement with the thermographic measurements shown in the inset, which indicate surface temperatures of 28.0 °C on the exposed side and 32.9 °C on the forehead side (Δ*T* = 4.9 K). The consistency between the thermographic and voltage-derived temperature differences confirms that the device reliably captures the environmental thermal gradient. Similar modest temperature differences have been reported in textile-integrated thermoelectric devices, where thermal coupling between the skin and textile substrate governs the effective gradient [59].

Figure 9b shows the output voltage and maximum power measured indoors at 28 °C when the wind at an airflow rate of 3 m/s was applied after 45 s. The inset shows an image depicting the TEG in operation during the measurement. The output voltage and maximum power rapidly increased and stabilized at 3.4 mV and 2.7 nW, respectively, corresponding to approximately 2.4-fold and 5.4-fold enhancements compared with the windless condition. This enhancement arose from forced convection, which increased heat dissipation from the outer surface and thereby enlarged the temperature gradient. These results demonstrate the strong influence of environmental heat-transfer conditions on the performance of wearable TEG.

Figure 9c shows the output voltage and maximum power measured outdoors at 7 °C with minimal wind. Although fluctuations of ±0.5 mV and ±2 nW were observed due to atmospheric variations, the device generated an average voltage of 6.1 mV and a maximum power of 9.0 nW, corresponding to approximately 4.4-fold and 18-fold enhancements compared with the indoor windless condition. The estimated temperature difference was 17.2 K, which is consistent with the thermographic measurements shown in the inset, where the exposed surface was 7.3 °C and the forehead-side region was 22.9 °C (Δ*T* = 15.6 K). When comparing the highest performance in this study with state-of-the-art reports on flexible TEGs using SWCNT/poly(aniline-co-acrylonitrile) composites at a similar temperature difference, the output voltage obtained here (6.1 mV) exceeds that of the state-of-the-art devices (4.1 mV), whereas the maximum power achieved in this study (9.0 nW) remains lower than the reported value (40 nW) [60]. These comparisons highlight that, although the voltage output of our device is competitive with leading reports, its power generation remains limited, which has important implications for practical operation. Because typical booster circuits require at least 5 mV to operate [61], the wearable TEG can power IoT sensors only under favorable environmental conditions such as cold outdoor temperatures or strong airflow. Improving performance will require increasing the internal temperature difference by modifying the device structure. Potential strategies include forming integrated *p*–*n* junction SWCNT films, introducing three-dimensional architectures to enhance thermal isolation, or incorporating low-thermal-conductivity spacer layers to suppress heat leakage [62,63,64,65]. These improvements could also support future applications such as heat-flux sensing and noninvasive physiological monitoring, where the rapid thermal response and high sensitivity of SWCNT-based films are advantageous [66,67,68,69]. Although further optimization is necessary, the present results demonstrate the feasibility of SWCNT-TEGs as wearable thermoelectric components. In addition to performance enhancement, future work will also require a systematic evaluation of TEG durability. Our previous study reported that repeated bending did not degrade the Seebeck coefficient of SWCNT films, although the electrical resistance increased after mechanical deformation [49]. As a result, the reduction in output voltage is expected to be small, whereas the maximum output power may decrease due to the increased resistance. Considering these findings, we plan to incorporate mechanical-durability requirements into the next stage of TEG development.

## 4. Conclusions

In this study, flexible TEGs were fabricated by dip-coating SWCNT films onto porous PPS meshes to develop lightweight and conformable power sources for wearable sensors. The dip-coated SWCNT/mesh films retained their mesh openings and mechanical flexibility while exhibiting stable *p*- and *n*-type thermoelectric properties. Their practical applicability was demonstrated in two representative wearable scenarios. First, a fingertip-responsive TEG composed solely of *p*-type films produced a rapid and nearly proportional increase in output voltage with the number of touching fingers, confirming its suitability as a self-powered tactile input element. Second, a *p*–*n* series-connected SWCNT-TEG sewn onto a cap generated 1.4 mV under indoor still-air conditions at 28 °C, and its output increased significantly under airflow or in a cold outdoor environment (7 °C), reflecting the strong influence of environmental heat-transfer conditions on device performance. These results demonstrate that dip-coated SWCNT/mesh-based TEGs are promising candidates for flexible and integrable power sources in wearable sensing systems. Further improvements in device architecture, such as enhancing thermal isolation or engineering advanced *p*–*n* structures, will be essential to increase the temperature difference across the device and achieve practical power levels for broader wearable applications.

## Figures and Tables

**Table 1:** Thermoelectric properties of dip-coated SWCNT/mesh films. The thermal conductivity values reported for the SWCNT/mesh films represent the effective thermal conductivity.

Sample	*S* [μV/K]	*σ* [S/cm]	*PF* [μW/(m·K^2^)]	*κ* [W/(m·K)]	Ref.
*p*-type dip-coated SWCNT/mesh films	54.1	6.3	1.8	3.6	This work
*n*-type dip-coated SWCNT/mesh films	−36.0	10.4	1.4	6.0	This work
*p*-type SWCNT film without openings	56	32	9.7	7.3	[41]
*n*-type SWCNT film without openings	−55	12	3.6	0.6	[30]

## Data Availability

The data presented in this study are available upon request from the corresponding author.

## Supplementary Materials

The following supporting information can be downloaded at https://www.mdpi.com/article/10.3390/mi17010139/s1: Figure S1: Higher-magnification FE-SEM of *p*-type dip-coated SWCNT/mesh film. Figure S2: Experimental setup and thermographic images used for effective thermal conductivity measurements via the comparative cut-bar method. Figure S3: Schematic diagram of the computational system used in the CFD simulations, including the geometrical configuration of the films and surrounding air domain, the boundary conditions applied at the heater–film interface, and the material properties employed in the calculations.
