# Supplementary material for: Flexible Mesh-Structured Single-Walled Carbon Nanotube Thermoelectric Generators with Enhanced Heat Dissipation for Wearable Applications
Source: Micromachines (Basel). 2026 Jan 22;17(1):139. doi: 10.3390/mi17010139 (PMC12843747; doi:10.3390/mi17010139)
Supplement: Supplementary file 1 [file micromachines-17-00139-s001.zip › micromachines-4100048-supplementary.pdf]

Flexible Mesh-Structured Single-Walled Carbon Nanotube Thermoelectric Generators with Enhanced Heat Dissipation for Wearable Applications

Hiroto Nakayama[1], Takuya Amezawa[1], Yuta Asano[2], Shuya Ochiai[1], Keisuke Uchida[1], Yuto Nakazawa[1], Masayuki Takashiri[1,2,*]

[1] Department of Materials Science, Tokai University, Hiratsuka, Kanagawa 259-1292, Japan

[2] Department of Applied Chemistry, Tokai University, Hiratsuka, Kanagawa 259-1292, Japan

* Corresponding author. Email: takashiri@tokai.ac.jp (Masayuki Takashiri)

Supplementary Materials

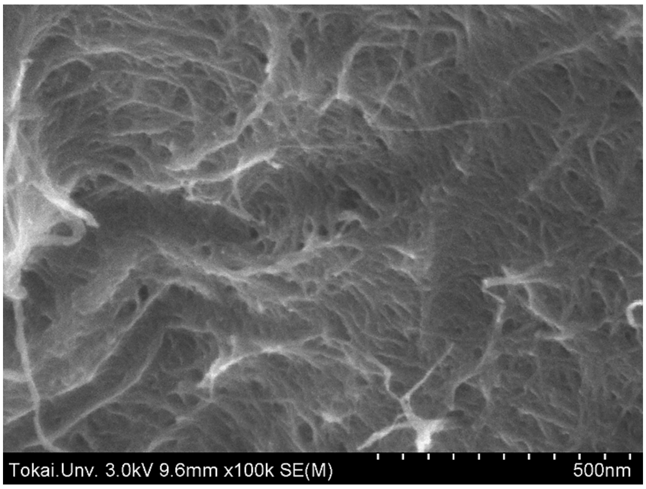

Figure S1. Higher-magnification FE-SEM of *p*-type dip-coated SWCNT/mesh film.

To visualize the SWCNT network more clearly, a higher-magnification FE-SEM image is shown in Figure S1.

Sample	Configuration	Thickness
(a)	*p*-type SWCNT mesh/film	70 μm
(b)	*n*-type SWCNT mesh/film	70 μm
(c)	*p*-type SWCNT film without openings	70 μm

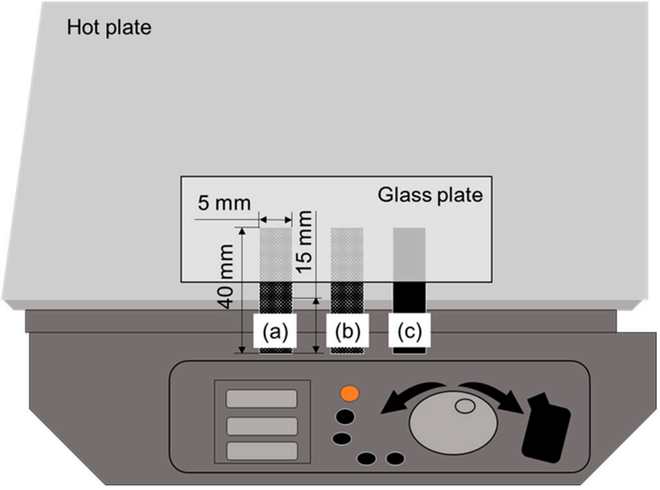

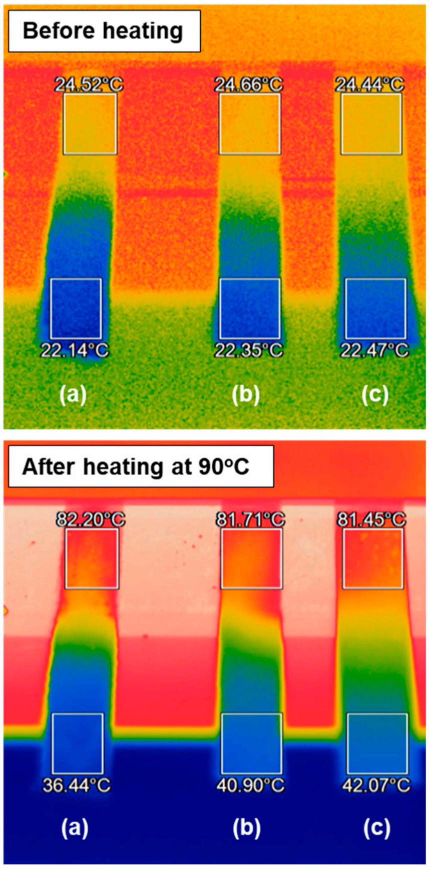

Figure S2. Experimental setup and thermographic images used for effective thermal conductivity measurements via the comparative cut-bar method.

To estimate the effective thermal conductivity of SWCNT mesh/films, the comparative cut-bar method was employed, as shown in Figure S2. In this method, the unknown sample and a reference material with known thermal conductivity are placed in series under identical heat flow conditions, and the thermal conductivity of the unknown sample is determined by comparing the measured temperature gradients. The unknown samples (*p*- and *n*-type SWCNT mesh/films) and the reference sample (dense *p*-type SWCNT film

without openings) were placed flat on the heater surface. Each film was partially covered and fixed by a glass plate, ensuring firm contact between the film and the heater. Approximately half of each film was in direct contact with the heater under the glass plate, while the remaining portion extended beyond the heated area. Prior to heating, the surface temperatures of the three samples exhibited no significant differences. At a heater temperature of 90°C, the temperature differences across the films were measured to be 45.76 K for the *p*-type SWCNT mesh/film, 40.81 K for the *n*-type SWCNT mesh/film, and 39.38 K for the dense *p*-type SWCNT film without openings. When the film thickness (d) differs among the samples, the relationship between thermal conductivity (κ) and the measured temperature difference (ΔT) under a constant heat flux (q) is given by: $q = \kappa A(\Delta T/d)$, where A is the cross-sectional area of the film. In this case, the temperature difference depends not only on the thermal conductivity but also on the film thickness: $\Delta T \propto d/\kappa$. Thus, for samples with larger thickness, the temperature difference increases even if the thermal conductivity is the same. Conversely, thinner films exhibit smaller temperature differences under identical heat flux conditions. The reference sample, a dense *p*-type SWCNT film without openings, exhibited a thickness of 70 μm and a thermal conductivity of 7.3 W/(m·K), as determined by the noncontact laser-spot periodic-heating radiation calorimetry method. The *p*-type and *n*-type SWCNT

mesh/films, with thicknesses of 40 μm and 60 μm, respectively, were evaluated using the comparative cut-bar method, yielding effective thermal conductivities of 3.6 W/(m·K) and 6.0 W/(m·K).

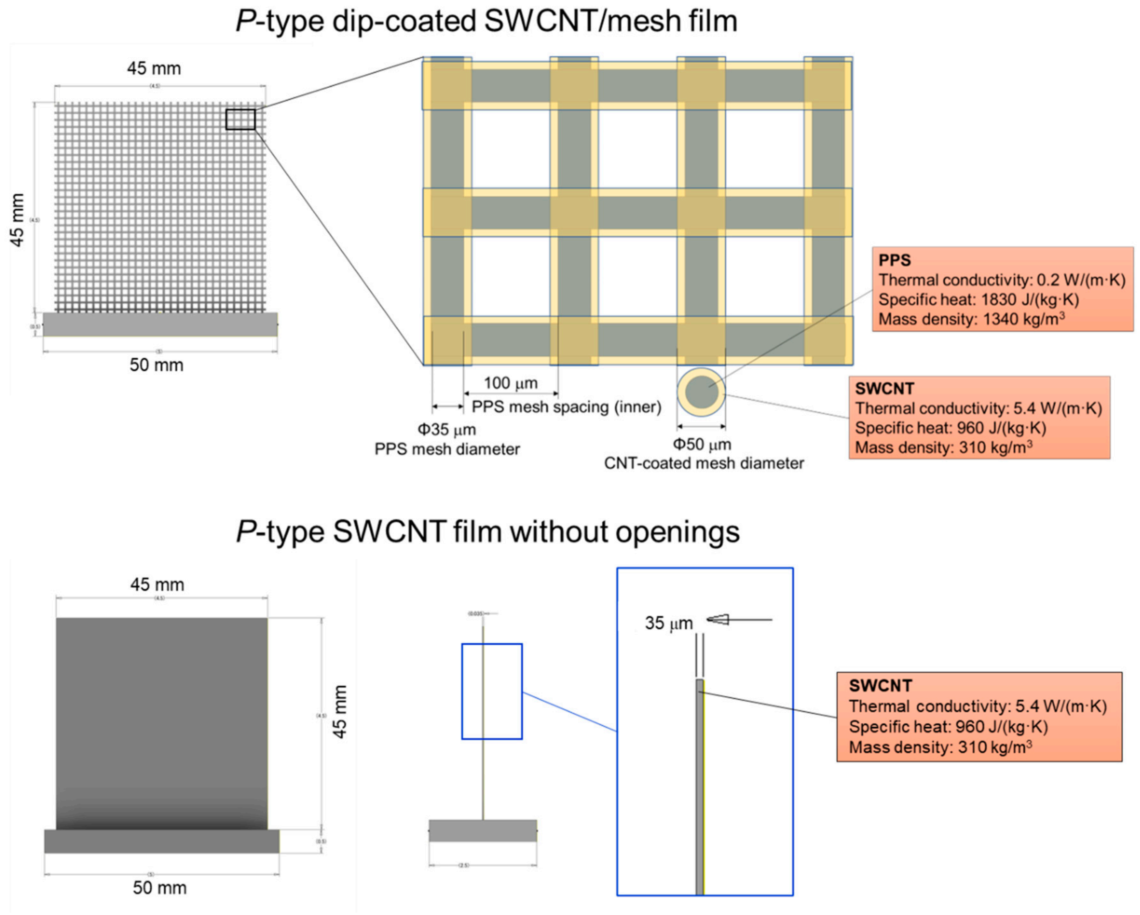

Figure S3. Schematic diagram of the computational system used in the CFD simulations, including the geometrical configuration of the films and surrounding air domain, the boundary conditions applied at the heater–film interface, and the material properties employed in the calculations.

To compute the temperature distribution in the *p*-type dip-coated SWCNT/mesh film and the *p*-type SWCNT film without openings, steady three-dimensional model based on computational fluid dynamics (CFD) was employed. The simulations were carried out

using an unstructured grid and solved with the finite volume method implemented in the commercial CFD code SCRYU/Tetra V14. Figure S3 shows the schematic diagram of a system used for the calculations. The heat transfer in the models of the films and the air atmosphere were governed by the conservation equations of mass, momentum, and energy. A standard k-ε turbulence model was used to model the airflow. The temperature of the contact surface between the heater and films was constant at 60°C. The geometries and physical properties of the material used in the calculations are described in Figure S3.
